# Plant bioactive compounds as functional feed additives for improving livestock and poultry production: review

**DOI:** 10.5713/ab.250961

**Published:** 2026-06-24

**Authors:** Do Thi Cat Tuong, Adhimoolam Karthikeyan, Lin Lat La Min, Sureshbabu Anjana, Masayuki Funaba, Sanggun Roh, Taesun Min

**Affiliations:** 1 Department of Animal Biotechnology, Jeju International Animal Research Center, Sustainable Agriculture Research Institute, Jeju National University, Jeju, Korea; 2 Jeju International Animal Research Center, Jeju National University, Jeju, Korea; 3 Division of Applied Biosciences, Graduate School of Agriculture, Kyoto University, Kyoto, Japan; 4 Graduate School of Agricultural Science, Tohoku University, Sendai, Japan

**Keywords:** Antibiotics, Phytogenic Feed Additives, Ruminant and Monogastric Nutrition, Sustainable Livestock Production

## Abstract

The administration of antibiotics to enhance growth and prevent diseases in livestock and poultry has elicited concerns over antimicrobial resistance and its implications for public health. The escalating demands on the livestock and poultry sectors necessitate enhanced productivity, diminished economic losses, and the guarantee of food safety for human consumption. Plant bioactive compounds (PBCs) have developed as safe and sustainable alternatives to antibiotics that enhance growth, bolster animal health, and boost productivity without jeopardizing food safety. PBCs represent a diverse group of secondary metabolites—including phenolics, terpenoids, polysaccharides, and organosulfur compounds—that exhibit a wide range of biological activities relevant to livestock and poultry nutrition and health. Recent studies have shown that supplementing animals with PBCs as feed additives, either as crude extracts or individual compounds, improves growth performance, nutrient utilization and modulates gut microbiota, while exerting antioxidant, anti-inflammatory, antimicrobial, and immunoregulatory effects, and thereby enhancing overall resilience of animals. The multifunctional properties of PBCs, along with the reduced risk of resistance development, position them as promising candidates for next-generation feed additives for livestock and poultry production. However, challenges remain regarding optimal concentration, variability in bioavailability, stability during feed processing, and the standardization of active components. Here, we discuss the major PBCs and their potential as functional feed additives to improve livestock and poultry production. We further highlight research gaps and outline future prospects required to promote their integration into sustainable animal production.

## INTRODUCTION

Antibiotics were initially introduced to treat infections in livestock and poultry, were later found to promote weight gain, which led to their widespread use as growth promoters in livestock and poultry farming over the past decades. However, the prolonged use of antibiotics in animals has raised significant concerns, including the emergence of antimicrobial resistance, the disruption of gut microbiota, and the potential transfer of resistant pathogens to humans through the food chain [1,2]. In recent years, growing awareness of the risks associated with antibiotic overuse has influenced consumer preferences. Modern consumers increasingly demand food products that are safe, natural, and free from antibiotic residues, driving the livestock and poultry industries to adopt more sustainable production practices [3]. In addition to consumer awareness, government regulations and policy interventions have played a pivotal role in promoting the reduction of antibiotic dependence in livestock and poultry production [4]. Many countries, particularly within the European Union, have already banned antibiotics as growth promoters, with similar restrictions being progressively implemented worldwide [5]. These challenges have driven a global search for safe and sustainable alternatives to antibiotics.

Plant-derived bioactive compounds (PBCs)—including phenolics, terpenoids, polysaccharides, and organosulfur compounds (OSCs)—have gained considerable attention as natural growth promoters and health modulators in livestock and poultry production. PBCs are naturally occurring secondary metabolites of plants that influence biological processes in living organisms, providing benefits beyond their basic nutritional functions [6,7]. Their multifaceted properties, including antioxidant, anti-inflammatory, antimicrobial, and immunomodulatory activities [8], make them promising candidates to replace or reduce the reliance on antibiotics and conventional feed additives in livestock and poultry farming. The beneficial effects of PBCs include the protection of dietary proteins from excessive degradation through the activity of tannins or polyphenol oxidase, which lowers soluble nitrogen levels and enhances nitrogen utilization efficiency in animals [9]. These compounds also contribute to reducing pollutant emissions, such as methane, while enhancing overall animal health and resilience. PBCs are increasingly recognized for their inherent benefits, characterized by reduced toxicity, low residue formation, a reduced risk of drug resistance development and enhanced safety [10]. However, some PBCs—even those considered beneficial at optimal levels—can exert anti-nutritional or toxic effects when present at inappropriate concentrations. Despite extensive research on PBCs in livestock and poultry nutrition, their efficacy and use remain inconclusive and limited. Addressing knowledge gaps requires interdisciplinary efforts to achieve economically viable, environmentally sustainable and eco-friendly animal production. In this review, we first discuss major PBCs, their sources, and mechanisms of action, followed by the potential of PBCs to improve animal performance, nutrition, health and product quality in livestock and poultry.

We further discuss existing research gaps and outline future perspectives necessary for their effective application in sustainable animal production. Relevant literature was collected from Web of Science, Scopus, and PubMed using keywords related to PBCs and livestock and poultry production. Priority was given to peer-reviewed, recent, and high-quality studies relevant to animal health and productivity.

## PLANT-DERIVED BIOACTIVE COMPOUNDs: CLASSIFICATION AND SOURCES

PBCs are broadly categorized into phenolic compounds, terpenoids, polysaccharides, and OSCs. These compounds possess distinct structural characteristics and exhibit diverse biological activities, including antioxidant, anti-inflammatory, antimicrobial, immunomodulatory, and growth-promoting activities, positioning them as promising alternatives to conventional feed additives and antibiotics. In animal nutrition, they are supplied as feed additives either in the form of crude extracts or as individual compounds. Phenolic compounds represent a large and diverse group of plant-derived metabolites found abundantly in fruits, vegetables, cereals, legumes, and nuts [11]. The main phenolic compounds include phenolic acids, flavonoids, stilbenes, lignans, and tannins. Owing to their structural diversity, these compounds exhibit a wide-range of biological activities and contribute to improved animal health and performance [12]. Similarly, terpenoids represent the largest and most structurally diverse class of secondary metabolites. They are derivatives of terpenes, containing oxygen functional groups and are structurally composed of five-carbon (C5) entities called isoprene units [13]. These compounds are distinguished by their core carbon skeletons and associated functional groups. Accordingly, terpenoids are classified in to several types including monoterpenoids, sesquiterpenoids, diterpenoids, triterpenoids, and polyterpenoids. Medicinal and aromatic plants are major sources of these compounds, which are often found in essential oils (EOs) and contribute to improved nutrient absorption, as well as antimicrobial and antioxidant activities [14]. Alongside phenolics and terpenoids, polysaccharides are structurally diverse carbohydrates that perform various biological functions, serving as structural components, energy reserves, and dietary fibers.

Polysaccharides are recognized for their positive impact on animal health by modulating inflammation, metabolism, and oxidative stress [15]. Major plant-derived polysaccharides include starch (abundant in grains and roots), cellulose (the principal structural polymer in plant cell walls), and pectin (prevalent in fruits and vegetables). Owing to their biodegradability, biocompatibility, and modifiable functional properties, plant polysaccharides are widely used in the food, pharmaceutical, and livestock industries. In addition to these compounds, OSCs represent another important group of PBCs that are abundantly present in Allium species such as garlic, onions, and leeks, as well as in cruciferous vegetables including broccoli, cabbage, and cauliflower [16]. Representative OSCs, such as allicin and sulforaphane, contribute to the characteristic pungent aroma of these plants and exhibit potential therapeutic properties. These compounds possess various biological activities, including antioxidant and anti-inflammatory activities and have shown potential to improve the animal performance by modulating microbiota in pigs [17]. Similarly alkaloids and glycosides represent another important group of PBCs that exhibit diverse biological activities. However, their efficacy and safety as feed additives depend largely on their specific chemical structures and concentrations. Alkaloids are nitrogen-containing compounds biosynthesized from amino acid precursors, particularly lysine, phenylalanine, tyrosine, and tryptophan, and they typically contain one or more nitrogen atoms within a heterocyclic ring [18]. Glycosides are compounds in which a sugar moiety (glycone) is linked via a glycosidic bond to the anomeric carbon of a non-sugar moiety (aglycone) [19]. Certain alkaloids (i.e., sanguinarine, berberine, and capsaicin) and glycosides, (i.e, saponins) have been reported to improve growth performance and gut health by regulating the intestinal microbiota, reducing methane emissions and improving nutrient utilization in animals [20–23]. However, some alkaloids and glycosides may exert toxicological and anti-nutritional effects at elevated doses; therefore, their application must be carefully managed.

## A BRIEF ACCOUNT ON MECHANISMS UNDERLYING THE EFFECTS OF PLANT-DERIVED BIOACTIVE COMPOUNDs

**Figure 1:**
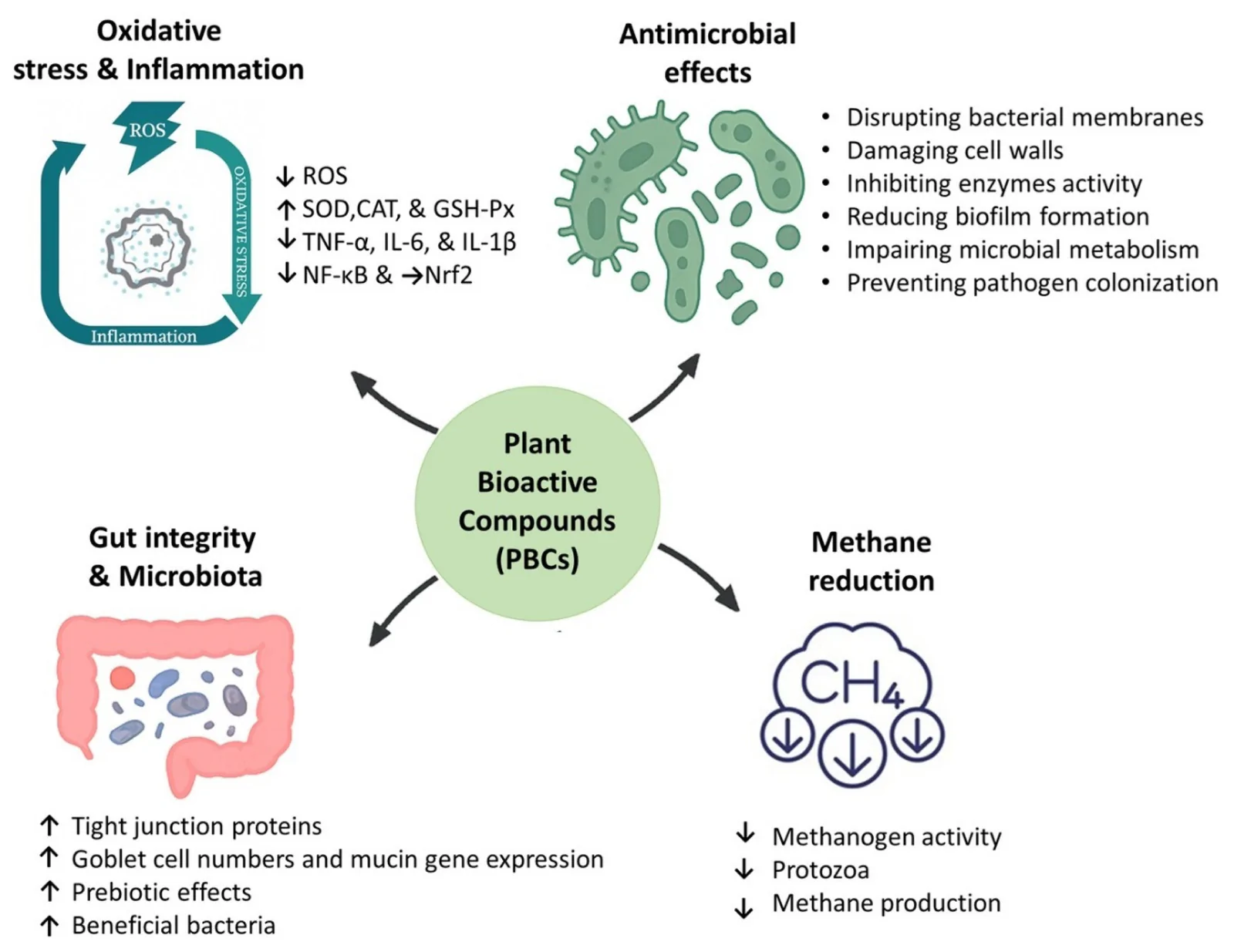
Mechanism underlying the beneficial effects of plant bioactive compounds. ROS, reactive oxygen species; SOD, superoxide dismutase; CAT, catalase; GSH-Px, glutathione peroxidase; TNF-α, tumor necrosis factor-alpha; IL-6, interleukin-6; IL-1β, interleukin-1 beta; NF-κB, nuclear factor kappa B; Nrf2, nuclear factor erythroid 2-related factor 2; CH_4_, methane.

PBCs are administered via animal feed either as crude plant extracts or as isolated bioactive compounds, depending on formulation and intended function. They regulate the oxidative stress, inflammation, microbial, metabolic, and stress-response pathways to enhance health and performance in livestock and poultry animals (Figure 1). Phenolic compounds, including flavonoids, tannins, and phenolic acids, act as direct radical scavengers, induce endogenous antioxidant enzymes (i.e., superoxide dismutase [SOD] and glutathione peroxidase [GSH-Px]), reduce pro-inflammatory mediators (i.e., interleukin [IL]-6), and regulate oxidative stress- and inflammation-associated pathways, including activation of nuclear factor erythroid 2-related factor 2 (Nrf2) and inhibition of nuclear factor kappa B (NF-κB) signaling [24–26]. These activities assist to improve oxidative balance and decrease chronic inflammation, both of which are major causes of impaired metabolic efficiency and increased disease susceptibility in animals. Niu et al. [27] demonstrated that supplementation with plant essential oil (PEO) or coated plant essential oil (CEO) enhances growth performance in nursery piglets and mitigates LPS-induced hepatic oxidative stress via activation of the SIRT1–PGC-1α signaling axis. Notably, CEO exhibits a more pronounced antioxidant effect than PEO. Beyond their antioxidant and anti-inflammatory effects, PBCs also exert antimicrobial effects through multiple mechanisms, including disruption of bacterial membranes, damage to cell walls, inhibition of major enzymes (DNA gyrase and topoisomerase), suppression of biofilm formation, impairment of microbial metabolism, and prevention of pathogen colonization [28–31]. Alongside their established therapeutic effects, PBCs contribute to the maintenance of intestinal barrier integrity and support efficient nutrient transport within the gastrointestinal tract (GI). These effects are mediated through multiple mechanisms, including regulation of the mRNA expression of tight junction (TJ) proteins, cytokines, and chemokines, increased goblet cell numbers and mucin gene expression, and regulation of intestinal immune response [10]. Collectively, these processes reduce passive intestinal permeability and strengthen epithelial barrier function. Notably, the mechanisms underlying PBCs mediated enhancement of TJ integrity vary depending on the specific compound. In addition, PBCs exhibit prebiotic-like effects by reshaping the gut microbiota, promoting beneficial non-pathogenic bacteria while suppressing pathogenic species through complex microbial interactions, thereby contributing to overall gut health.

Mao et al. [32] reported that supplementation with quercetin alleviated weaning-associated diarrhea by regulating the NF-κB signaling pathway and controlling the balance between anti-inflammatory and pro-inflammatory factors. Additionally, quercetin reshaped the gut microbiota by reducing Proteobacteria and enhancing beneficial bacterial diversity, thereby improving intestinal homeostasis, nutrient absorption, feed efficiency, and overall growth performance in weaned piglets. Supplementation with PBCs and crude plant extracts has been shown to reduce methane emissions through multiple mechanisms. Methane production in ruminants is primarily driven by methanogenic archaea that use major fermentation end products as substrates. Dietary inclusion of PBCs has been reported to suppress members of the *Methanobacteriaceae*, a dominant methanogenic family thereby decreasing methane level. An important mechanism involves the inhibition of ruminal protozoa. Hydrogenosomes with in ruminal protozoa generate H_2_, which serves as a primary substrate for methanogenesis through the hydrogenotrophic methanogenesis [33]. Consistently, a strong positive association among protozoa abundance and methane generation has been reported [34]. For instance, supplementation with mulberry leaf flavonoids (MLF) [35] reduced the number of protozoa population and consequently decreased methane production.

## PLANT-DERIVED BIOACTIVE COMPOUND-DRIVEN EFFECTS IN FARM ANIMALS HEALTH AND PRODUCTION

### Effects of plant-derived bioactive compound supplementation in poultry

Flavonoids have been associated with numerous biological benefits in poultry, including improved growth performance, anti-microbial activity, and enhanced antioxidant activity. Owing to their strong antioxidant properties, dietary flavonoids are widely explored as functional feed additives to promote poultry health and productivity. Supplementation with flavonoids such as genistein [36], hesperidin [36,37], quercetin [38], naringin [37], baicalin [24], and rutin [39] have enhanced total antioxidant capacity, thereby strengthening the endogenous defense system against oxidative damage. Among these compounds, genistein and hesperidin downregulate heat shock protein expression—a molecular marker of cellular stress [36]. In addition, hesperidin and naringin reduce malondialdehyde concentrations, a widely recognized indicator of lipid peroxidation [37]. Collectively, these effects contribute to the attenuation of oxidative stress, preservation of cellular integrity, and improved physiological performance in poultry. Quercetin act as a phytogenic feed additive in poultry, improving growth performance and meat quality through multiple mechanisms. These include inhibition of low-density lipoprotein oxidation, protection of erythrocytes from oxidative damage, and broad-spectrum antibacterial activity against *Salmonella enterica* serotype Typhimurium, *Escherichia coli*, *Staphylococcus aureus*, and *Pseudomonas aeruginosa* in broiler chickens [38,40].

Curcuminoids and lipophilic turmeric extracts, which contain bioactive constituents such as curcumin and turmerones, possess well-documented gastroprotective, antioxidant, and anti-inflammatory properties. These activities contribute to improved intestinal integrity and more efficient nutrient utilization in poultry. Dietary inclusion turmeric-derived compounds has therefore been associated with enhanced the growth performance in broiler chickens [41]. Consistently, Rajput et al. [42] reported that supplementation of broiler diets with curcumin for 42 days considerably increased body weight gain and feed efficiency during the finishing stage (22–42 days), although no considerable improvements were observed during the starting period (0–21 days). In addition to its growth-promoting effects, curcumin exhibits hepatoprotective properties by mitigating the toxic effects of aflatoxins, primarily through inhibition of cytochrome P450 isozymes in the liver [43]. Similarly, Sun et al. [44] demonstrated that dietary lycopene supplementation (0–80 mg kg^−1^ feed) improved reproductive performance in laying hens by strengthening both hepatic and systemic antioxidant defenses. Lycopene increased the activities of SOD, T-AOC, glutathione peroxidase, and GSH/GSSG ratio, while simultaneously lowering serum cholesterol levels and increasing HDL-cholesterol and triiodothyroxine levels. These physiological changes, collectively improved oxidative stability and reproductive efficiency in hens. Dietary supplementation with *Lycium barbarum* flavonoids (500 mg/kg) has been shown to improve growth performance, intestinal barrier function, immune responses, and antioxidant capacity is ducks. These beneficial effects were associated with activation of the Nrf2 signaling pathway and inhibition of the of the NF-κB pathway, suggesting a regulatory role in oxidative stress and inflammatory responses [45]. Similarly, Hong et al. [46] reported that the inclusion of EO (125 ppm) derived from oregano, anise, and citrus peel in broiler diet improved overall performance. Birds receiving the supplemented diet exhibited approximately ~10% higher survival rates along with reduced serum cholesterol and very low density lipoprotein, indicating improved physiological status and metabolic health.

EO have also been reported to improve meat quality in poultry, producing more tender breast meat and juicier thigh muscles compared with unsupplemented controls. In addition, dietary inclusion of EO containing bioactive compounds in menthol, anethole, and eugenol has been associated with improved growth performance in broiler chickens [47]. Similar performance-enhancing effects have been documented for carvacrol administered either alone [48] or in combination with thymol [49]. Beneficial impacts on production traits have also been observed in laying hens supplemented with thymol and cinnamaldehyde [47] or with EO derived from *Citrullus lunatus* [50]. Beyond their growth promoting properties, EO and trans-cinnamaldehyde exhibit strong antimicrobial activity in poultry. *Salmonella* infection represents a major concern in the poultry industry; however, dietary cinnamaldehyde supplementation has been shown to effectively reduce bacterial colonization, limit horizontal transmission, and decrease egg contamination in both broiler and layer chickens [51].

Dietary supplementation with *Astragalus* polysaccharides (APSs) has been shown to enhance intestinal mucosal immunity in Newcastle disease–vaccinated chickens. APS administration increased the jejunal villus height-to-crypt depth ratio, elevated the IgA^+^ cell abundance, and enhanced secretory IgA levels, collectively indicating improved intestinal immune function. These findings suggest that APS can serve as an effective natural immunopotentiator and vaccine adjuvant in poultry [52]. Similarly, dietary inclusion of polysaccharides from *Ginseng*, APS, and *Salvia miltiorrhiza* reported to improve growth performance and immune competence in broiler chickens. These polysaccharides enhance serum immunoglobulin levels, regulate cytokine production, increase antioxidant enzyme activities, promote volatile fatty acids, and beneficially modulate the composition of the cecal microbiota [15]. Onion contains a range of bioactive compounds, particularly OSCs, which have been associated with several physiological benefits in poultry. Dietary supplementation with onion derived-OSCs has been reported to reduce blood glucose levels, potentially stimulating the central nervous system and increasing feed intake, thereby contributing to improved body-weight gain [53]. Inaddition, Jimoh et al. [54] observed that dietary garlic supplementation, across various inclusion levels, significantly decreased the caecal population of *Clostridium perfringens* compared with the control group, an effect attributed to its OSC content. Isoquinoline alkaloids have also demonstrated beneficial effects in broiler production systems. These compounds effectively alleviated stress induced by high stocking density, improving growth performance, immune responses, and intestinal health. Dietary supplementation with isoquinoline alkaloids derived from *Macleaya cordata* (0.6 mg/kg) alleviated lipopolysaccharide-induced liver injury by enhancing immune function and suppressing hepatic inflammation through modulation of the TLR4/MyD88/NF-κB signaling pathway [55]. Similary, supplementation with isoquinoline alkaloids and flavonoids, alone or in combination, alleviated heat and density stress in broilers by improving growth, gut morphology, and anti-inflammatory responses. Their combined application showed synergistic effects by improving microbiota diversity and metabolic pathways, demonstrating potential as effective feed additives under challenging rearing conditions [56]. The effects of the various PBCs in poultry are presented in Table 1.

### Effects of plant-derived bioactive compound supplementation in livestock animals

*Ruminants:* Dietary supplementation with quercetin in beef cattle has shown limited effects on meat quality and antioxidant activity. Although inclusion of 42 ppm quercetin increased loin pH and cohesiveness, no significant improvements were observed in antioxidant capacity, lipid oxidation, or other physicochemical characteristics. These findings suggest that a low inclusion level of quercetin (10%) may be insufficient to produce measurable biochemical benefits in beef production systems [57]. *In vitro* investigations have demonstrated that flavonoids such as naringin and quercetin, when supplemented at 4.5% of substrate, dry matter, effectively suppressed methane production without negatively affecting rumen fermentation efficiency. While, other flavonoids, including flavone, myricetin, catechin, rutin, and kaempferol, were found to reduce dry matter degradability, enzyme activities, and microbial growth [58]. Supplementation with MLF and resveratrol has also been reported to improve nutrient digestibility and reduced methane emissions in sheep. Both polyphenols enhanced energy utilization and decreased CH_4_ and CO_2_ production, with resveratrol showing comparatively stronger mitigation effects. However, neither treatment improved nitrogen retention, indicating selective benefits related to methane mitigation and feed efficiency [59]. Consistent with these findings, Ma et al. [35] reported that supplementation improved nutrient digestibility and modulated rumen microbial population in sheep. Flavonoids increased nitrogen and fiber digestibility, elevated total volatile fatty acids, and reduced methane emissions by suppressing protozoa and methanogen while promoting the abundance of *Fibrobacter succinogenes*, thereby enhancing rumen efficiency and decreasing methanogenesis.

Dietary supplementation with flavonoids derived from *Allium mongolicum* Regel has been shown to improve growth performance and regulate neuroendocrine function in meat sheep. Inclusion levels ranging from 11–33 mg/kg increased average daily gain and feed intake, while reducing the feed conversion ratio. These responses were accompanied by elevated serum concentrations of GH and IGF-1 levels, along with decreased corticosterone, indicating improved growth efficiency and enhanced stress resilience through endocrine regulation [60]. Similarly, flavonoids extracted from *Citrus aurantium* have been reported to influence feeding behavior and rumen health in Holstein bulls. Although no significant changes in overall growth performance was observed, animals receiving flavonoid supplementation exhibited smaller meal sizes, reduced agonistic and sexual behaviors, and healthier rumen walls. These findings suggest that citrus-derived flavonoids may enhance animal welfare and gastrointestinal resilience in cattle [61]. Dietary supplementation with citrus flavonoids, particularly hesperidin and naringin, has been reported to enhance milk oxidative stability of milk in dairy ewes without affecting milk yield, composition, or fatty acid profile. These bio active compounds reduced milk malondialdehyde levels, indicating improved antioxidant protection and enhanced resistance to oxidative deterioration, while maintaing milk quality or coagulation properties [62]. Citrus flavonoids also exhibit synergistic potential in regulating rumen fermentation. *In vitro* studies have shown that naringin and hesperidin, either supplemented together or as part of a mixed citrus flavonoid extract (CFE), more effectively inhibited ruminal methanogenesis and ammoniagenesis. This effect was associated with suppression of methanogenic archaea (*Methanobrevibacter*) and rumen protozoa (*Isotricha*, *Entodinium*), thereby improving nitrogen utilization and reducing enteric methane (CH_4_) emissions in dairy cows [63].

More recently, Hao et al. [64] reported that sea-buckthorn flavonoids improved growth performance, nutrient digestibility, and antioxidant status in finishing lambs. Moderate inclusion level enhanced rumen fermentation, stimulated microbial protein synthesis, and improved nitrogen utilization, while simultaneously reducing oxidative stress markers. These results suggest that sea-buckthorn flavonoids can enhance metabolic efficiency and oxidative resilience in ruminant production systems. Twelve Liuyang black goats were assigned to control or 2% tannic acid diets in a two-stage experiment for 20 days. Tannic acid significantly reduced methane emissions and fiber intake (p<0.05). Microbial shifts included increased *Firmicutes* and reduced *Methanobrevibacter* and *Prevotella*. Rumen fermentation was altered, with increased valerate and butyrate and a reduced acetate-to-propionate ratio. These findings suggest tannic acid mitigates methane production by modulating rumen microbial composition and fermentation [65]. Similarly, dietary inclusion of tea saponins in Dorper× Thin-tailed Han ewes improved rumen fermentation characteristics, nutrient utilization, and methane mitigation. Saponin supplementation enhanced the digestibility of organic matter, nitrogen, neutral detergent fiber, and acid detergent fiber, while increasing nitrogen retention and reducing methane emissions by 8.8%. Additionally, it elevated the proportion of propionate and the abundance of *Fibrobacter succinogenes*, while decreasing ruminal ammonia concentration’s and protozoa populations, indicating improved rumen efficiency and reduced methanogenesis [66]. Yang et al. [67] reported that dietary supplementation of garlic EO (5 g/day) in the diets of lactating cows tended to enhance milk fat content (p<0.10) without significantly affecting dry matter intake or ruminal fiber digestibility. Similarly, Hundal et al. [68] evaluated several EOs, including cinnamaldehyde, carvacrol, limonene, and carvone to determine their efficacy in mitigating enteric methane emissions while maintaining nutrient digestibility. Although supplementation levels exceeding 1% of carvacrol or limonene effectively reduced methane production, these higher inclusion rates were also associated with a decline in overall nutrient digestibility.

In another study, dietary supplementation with orange peel EO (up to 300 mg/kg concentrate) to lactating Chios ewes improved milk yield as well as fat and ash production, while reducing the proportion of milk unsaturated fatty acids and the atherogenic index in milk. However, increasing the supplementation level to 450 mg/kg did not further enhance milk yield, although it improved antioxidant status in both plasma and milk [69]. Rapeseed oil contain sterols and phenolic acids, and its dietary inclusion of rapeseed oil at 41 g/kg of dry matter in the lactating dairy cows diets has been shown to reduce daily methane emissions by approximately 22.5%. This reduction was associated with shifts in rumen microbial composition, including increased abundance of *Methanosphaera* and members of the *Succinivibrionaceae* family, along with a decrease in *Bifidobacteriaceae* populations [70]. Similarly, supplementation with *Zanthoxylum bungeanum* EO influenced the composition of the small-intestinal microbiota in sheep. The treatment altered the relative abundance of key bacterial genera, including *Prevotella*, *Ruminococcus*, and *Christensenellaceae*, which are closely associated with nutrient digestion and intestinal health [71]. In another study, administration of oregano essential oil (OEO) at a dosage of 300 mg/kg enhanced the growth rate of Sewa sheep. The enhancement in growth was associated with improved intestinal morphology and beneficial modulation of gut microbiota composition. Specifically, OEO supplementation significantly increased average daily gain, slaughter rate, small intestinal villus length, and the abundance of beneficial gut bacteria [72]. Similarly, supplementation with *Lippia graveolens* EO in lambs did not significantly influence growth performance, rumen fermentation traits, or carcass yield. However, it enhanced meat antioxidant activity, improved crude protein stability, and extended meat shelf life, indicating improved meat quality without negatively affecting productive performance [73]. Also, Ritt et al. [74] investigated the effects of OEO supplementation (60 mg kg^−1^ body weight day^−1^) in pre-weaned Holstein calves. The supplementation increased starter feed intake and enhanced microbial diversity in both the rumen and jejunum. In addition, OEO reduced the abundance of potentially pathogenic genera, including *Streptococcus*, *Escherichia*, and *Clostridium*, indicating improved microbial balance within the GI. However, no significant effects were observed on overall growth performance or rumen fermentation parameters, suggesting that OEO primarily modulates gut microbiota and feed intake without markedly altering physiological growth responses.

APS is a predominant water-soluble heteropolysaccharide derived from the roots of *Astragalus membranaceus*. It mainly consists of monosaccharides such as glucose, arabinose, and galactose interconnected through glycosidic linkages. Dietary supplementation with *APS* has been reported to improve rumen fermentation characteristics and antioxidant status in weaned lambs. Supplementation enhanced growth performance, propionate production, increased antioxidant enzyme activities, and stimulated immune responses, while reducing plasma cortisol levels [75]. Supplementation enhanced growth performance and immune responses while improving rumen microbial composition in yaks, with increased abundance of Methanobrevibacter and Butyrivibrio, microbes involved in fiber degradation and immune regulation [76]. Also, it improved growth performance, rumen function, metabolic efficiency, and serum antioxidant capacity in Angus bulls [77]. Alfalfa and sea weed polysaccharides supplementation significantly reduced diarrhea in calves, with reductions of 18.12% and 30.9%, respectively. These effects were linked to modulation of gut microbiota, characterized by increased beneficial bacteria and decreased pathogens, along with regulation of immune and inflammatory pathways. Sea weed polysaccharides exhibited greater efficacy than Alfalfa polysaccharides in alleviating intestinal inflammation [78]. Garlic powder rich in OSCs has been shown to reduce enteric methane emissions and enhanced milk yield in lactating Murrah buffaloes without affecting nutrient digestibility or milk composition. Supplementation at 2% DMI lowered methane output by up to 31%, indicating garlic derived OSCs as natural feed additives for improving rumen efficiency and promoting sustainable milk production [79]. A summary of the diverse effects of PBCs in ruminant animals is presented in Table 2.

*Pigs:* Piglets fed diets enriched with polyphenol-rich extracts, such as grape or hop, exhibited reduced expression of inflammation-associated genes in the duodenum, ileum, and colon accompanied by a decreased abundance of *Clostridium* in the fecal microbiota [80]. Similarly, supplementation with polyphenol-rich seed and pomace extracts downregulated NF-κB signaling in the duodenal mucosa, indicating suppression of intestinal inflammatory responses [81]. In another study, piglets challenged with *E. coli* and fed commercial polyphenol-rich diets showed a reduced incidence of diarrhea, further confirming the intestinal anti-inflammatory potential of polyphenols [82]. In LPS-challenged piglets, dietary inclusion of soy isoflavones strengthen immune function while reducing diarrhea and circulating endotoxin levels [83]. Moreover, resveratrol supplementation effectively alleviated intestinal inflammation and oxidative stress induced by deoxynivalenol exposure, while improving microbial balance and intestinal barrier integrity in piglets [84]. Dietary supplementation with quercetin has been shown to enhance growth and immune function in growing pigs challenged with LPS. Specifically, quercetin improved average daily gain and nutrient digestibility, while modulating immune responses by decreasing the pro-inflammatory cytokine IL-6 and increasing immunological indicators such as IgG, white blood cell counts, and lymphocyte levels. These responses suggest that quercetin exerts immunomodulatory and growth-promoting effects under inflammatory conditions [25].

Seven days supplementation with puerarin markedly enhanced the intestinal antioxidant defense system in 18-day-old piglets challenged with porcine epidemic diarrhea virus. This improvement was evidenced by increased activities of antioxidant enzymes, including SOD and GSH-Px in the duodenum and colon. In addition, puerarin administration reduced the concentrations of oxidative stress indicators such as H_2_O_2_ and MDA levels in the ileum, indicating a protective effect against virus-induced oxidative damage in the intestinal tissues [26]. Recent studies have demonstrated that MFL enhanced growth performance, carcass traits, and antioxidant capacity in pigs. Dietary supplementation with MFL has been shown to improve the growth performance and meat quality, particularly by enhancing meat tenderness. Furthermore, MFL supplementation has been reported to reduce fat synthesis and favorably alter fatty acid profiles in adipose tissue, indicating its potential to improve lipid metabolism and overall meat quality in pigs [85,86]. Citrus flavonoids supplementation promoted piglet growth and intestinal health by regulating the TLR2/NF-κB signaling pathway. It improved gut microbial balance by increasing the relative abundance of Bacteroidetes and Prevotella, while reducing Tenericutes and Clostridiales, indicating a shift toward a more beneficial intestinal microbial community. An inclusion level of 80 mg/kg was identified as the most effective dose for improving piglet’s performance [87]. Gan et al. [88] demonstrated that dietary curcumin supplementation reduced the intestinal abundance of pathogenic *E. coli* in weaned piglets. This treatment also suppressed the TLR4 signaling pathway, modulated IL expression, and increased immunoglobulin levels, indicating improved intestinal immune responses. Similarly, Zhang et al. [89] reported that curcumin act as a natural antioxidant and can be incorporated into dietary strategies to alleviate oxidative stress in IUGR offspring, thereby improving redox status in leg muscle and meat quality. In addition, curcumin supplementation has been shown to attenuate oxidative damage and protect the structural integrity of jejunal cell organelles and membranes in piglets [90]. Brus et al. [91] reported that supplementation with a mixture of chestnut wood tannins and organic acids reduced harmful *E. coli* populations while increasing beneficial lactic acid bacteria in piglets during an 82–127 day feeding period. Similarly, dietary inclusion of EOs containing cinnamaldehyde (15%) and thymol (5%) combined with organic acids, improved growth performance, resulting in approximately 13.5% higher average daily gain and 5.6% greater final body weight in weaned piglets [92]. EO rich in thymol and cinnamaldehyde were also shown to reduce IL-6 production, enhance lymphocyte proliferation, and increase plasma IgA and IgM levels in weaning pigs. Moreover, EO containing cinnamaldehyde, thymol, and carvacrol reduced fecal nitrogen and ammonia emissions by suppressing microbial protease and urease activities [93,94]. Additionally, supplementation with 2,000 mg/kg EO coated with glycerol monolaurate in weanling piglets enhanced serum antioxidant capacity and immune status, modulates jejunal inflammatory cytokines and intestinal morphology, and thereby promotes improved growth performance [95].

Artuso-Ponte et al. [96] reported that supplementation with quaternary benzo[c]phenanthridine alkaloids derived from herbal extracts alleviated transportation stress in pigs and positively influenced pork safety. Achyranthes bidentata polysaccharide supplementation improved physiological functions in weaned piglets. A higher dose (1,600 mg/kg) increased serum phosphorus and supported hepatic health, whereas a lower dose (800 mg/kg) elevated iron and immunoglobulins. Both doses enhanced antioxidant capacity and improved lipid metabolism by reducing triglyceride and LDL cholesterol [97]. The combined supplementation of boric acid and plant polysaccharides synergistically enhanced growth performance and immune function in fattening pigs by increasing intestinal enzyme activity, antioxidant capacity, and nutrient absorption, while reducing emissions of harmful gas and heavy metals [98]. Moreover, *Achyranthes bidentate* polysaccharide, and glycyrrhiza polysaccharides have been shown to counteract porcine reproductive and respiratory syndrome virus infection [99,100]. Similarly, APS inhibits the proliferation of porcine circovirus type 2 and mitigates associated tissue damage [28]. A summary of the diverse biological effects of PBCs in non-ruminant animals is presented in Table 3.

## CONCLUSION AND AREAS OF FUTURE RESEARCH

PBCs influence livestock health and performance through diverse physiological and metabolic processes. Their effects depend on factors including botanical origin, chemical composition, bioavailability, dosage, and host interactions, while variability in composition complicates evaluation of efficacy and delivery strategies. Antioxidant, anti-inflammatory, antimicrobial, immunomodulatory, and microbiota-regulatory activities have been widely reported for PBCs; however, the underlying molecular mechanisms governing these effects remain incompletely understood. Therefore, future studies should prioritize mechanistic characterization and integrated omics approaches to clarify the molecular mechanisms governing PBCs activities. Beyond evaluating biological activities of PBCs, research should also consider economic feasibility, environmental impacts, and practical implementation in livestock and poultry systems. Assessing production costs, resource efficiency, and scalability of PBC-based feed additives will be essential to ensure that these strategies contribute to environmentally responsible and economically sustainable animal production.

Although most studies report beneficial effects, certain PBCs may produce adverse effects under specific conditions. These adverse effects are often dose-dependent, as excessive inclusion levels can impair feed intake, reduce nutrient digestibility, and disrupt normal organ function, showing the importance of carefully determining optimal dosage levels for safe and effective application in livestock and poultry nutrition. For instance, compounds that confer antioxidant and antimicrobial may also exhibit antinutritional properties at high concentrations by binding dietary proteins and reducing feed palatability. In addition, elevated levels of some PBCs have been reported to impair sperm function in reproductive contexts. Therefore, careful evaluation of the safety, dosage, and conditions of use of PBC-based feed additives is essential when assessing their suitability for animal production. Furthermore, establishing comprehensive and standardized evaluation system is essential for the reliable assessment of PBCs in livestock production. Moreover, many PBCs exhibit limited stability and are rapidly degraded in the GI, restricting their bioavailability and functional efficacy. To address this limitation, advanced delivery systems—including micro- and nanoencapsulation and lipid-based carriers—have been developed to enhance stability and enable targeted release. However, their large-scale application in animal feed remains constrained by challenges related to cost, scalability, and regulatory approval. Overcoming these barriers will require standardized formulations and coordinated efforts among researchers, industry, and regulatory agencies.

Plant based feeds have long been used in traditional animal farming practices on various forms; however, only in recent years has research intensified to elucidate biosynthesis, distribution, chemistry, and mechanisms of action of PBCs in animals. The integration of PBCs into livestock production offers a promising opportunity to develop sustainable, cost-effective, and environmentally friendly alternatives to conventional antibiotics. However, recognizing PBCs potential requires further research to evaluate their safety, efficacy, and consistency prior to large-scale implementation in commercial production systems.

## Figures and Tables

**Table 1:** The major biological effects of plant bioactive compounds in poultry

S.No	Plant bioactive compounds	Species	Doses	Biological effects	References
1	Quercetin	Chicken	1 g/kg of basel diet for 42 d	Improved health condition, growth performance and meat quality, inhibited low-density lipoprotein oxidation, protected erythrocytes from oxidative damage	[38]
2	*Lycium barbarum* flavonoids	Ducks	500 mg/kg of basel diet for 42 d	Improved growth performance, intestinal barrier function, immune response, and antioxidant capacity in ducks	[45]
3	Turmeric extracts, containing curcumin and turmerones (TF-36)	Chicken	1% of basel diet for 42 d	Contributed to improved gut integrity and nutrient utilization, and enhanced the growth performance.	[41]
4	Curcumin	Chicken	200 mg/kg of basel diet for 42 d	Enhanced body weight gain and feed efficiency during the finishing stage, although no marked effects were observed during the starting period.	[42]
5	Curcumin	Chicken	150 mg/kg of basel diet for 28 d	Curcumin protected the AFB1-induced liver injury, through the synergistic actions of increased antioxidant capacities and inhibition of the CYP450 isozyme	[43]
6	Lycopene	Chicken	20–80 mg/kg of basel diet for 35 d	Improved reproductive efficiency in hens by elevating hepatic and systemic antioxidant defenses. Increased SOD, T-AOC, glutathione peroxidase, and GSH/GSSG ratios, reduced serum cholesterol and elevated HDL-cholesterol and triiodothyroxine.	[44]
7	Isoquinoline alkaloids	Chicken	0.6 mg/kg of basal diet	Supplementation enhanced immunity, suppressed LPS-induced hepatic inflammatory cytokines and caspase activity, and downregulated genes associated with the TLR4/MyD88/NF-κB signaling pathway.	[55]
8	Essential oil/trans-cinnamaldehyde	Chicken	1% and 1.5% of basel diet for 49 d	Salmonella infection effectively controlled by cinnamaldehyde supplementation, which reduced bacterial colonization, horizontal extent, and egg contamination	[51]
9	*Astragalus* polysaccharides	Chicken	1–4 mg/mL	Enhanced intestinal mucosal immunity in newcastle disease–vaccinated chickens. Increased jejunal villus height-to-crypt depth ratios, IgA^+^ cell abundance, and secretory IgA levels, and improved gut immune responses.	[52]
10	Polysaccharides from *Ginseng*, *Astragalus* polysaccharide, and *Salvia miltiorrhiza*	Chicken	1,000 mg/kg of basel diet for 42 d	Improved the growth performance, strengthened the immune responses by elevating serum immunoglobulins and regulating cytokines, and increased antioxidant enzyme activity and volatile fatty acids	[15]
11	Organo sulfur compounds from onion	Chicken	1 % onion extract in drink water+basal diet	Lowered blood glucose, stimulated the central nervous system, and increased feed intake, collectively enhanced metabolic activity and promoted significant body-weight gain	[53]

SOD, superoxide dismutase; T-AOC, total antioxidant capacity; GSH, reduced glutathione; GSSG, glutathione disulfide; HDL, high-density lipoprotein; TLR4, toll-like receptor 4; MyD88, myeloid differentiation primary response 88; NF-κB, nuclear factor kappa B; IgA, immunoglobulin A.

**Table 2:** The major biological effects of plant bioactive compounds in ruminant animals

S.No	Plant bioactive compounds	Species	Doses	Biological effects	References
1	Quercetin	Beef cattle	42 ppm and 75 d	Slightly increased loin pH and cohesiveness but showed limited effects on antioxidant status and meat quality.	[57]
2	Hesperidin and naringin	Dairy ewes	6,000 mg/kg of feed for 28 d	Improved milk oxidative stability, reduced malondialdehyde levels, without altering milk yield, composition, fatty acid profile, or coagulation properties.	[62]
3	Citrus flavonoids	Holstein bulls	0.04% and 168 d	Improved rumen health, reduced stress behaviors, and modulated feeding without affecting overall growth performance.	[61]
4	Mulberry flavonoids and resveratrol	Sheep	2 g and 0.5 g and 8 d	Improved nutrient digestibility and energy use while reducing CH_4_ and CO_2_ emissions; resveratrol was more effective.	[59]
5	Tannic acid	Black goats	2% Tannic acid, g/kg diet and 20 d	Dietary tannic acid reduced methane emissions and improved feed efficiency by modulating rumen fermentation patterns and altering microbial community composition.	[65]
6	Tea saponin	Dorper cross bred ewe	2 g/d/head	Improved rumen fermentation and nutrient utilization, reducing methane by 8.8% and enhancing propionate and *Fibrobacter succinogenes* abundance and lowering ammonia and protozoa populations.	[66]
7	Oregano essential oil (OEO)	Lamb	0.02% and 0.04% of basel diet and 60 d	Did not affect growth, rumen traits, or carcass yield but enhanced meat antioxidant activity, crude protein stability, and shelf life, indicating improved meat quality without compromising performance.	[73]
8	*Astragalus membranaceus* polysaccharides	Lamb	15 g/kg of basel diet and 30 d	Enhanced rumen fermentation, antioxidant enzyme activity, and immunity, reducing cortisol without affecting growth or nutrient digestibility.	[75]
9	*Astragalus* polysaccharides	Yaks	1 g/kg of basel diet and 60 d	Enhanced growth performance and immune function and also optimized the rumen microbial composition. The relative abundance of Methanobrevibacter and Butyrivibrio, microbes associated with fiber degradation, anti-inflammatory activity, and immune regulation, was markedly increased.	[76]
10	Dietary coated *Astragalus* polysaccharide	Angus bulls	1.8 g/kg of dry matter and 60 d	Effectively improved growth performance, rumen functionality, metabolic efficiency, and serum antioxidant capacity in Angus bulls.	[77]
11	Alfalfa and sea weeds polysaccharides	Calves	4 g/calf/day with basal diet and 56 d	Alfalfa and sea weeds polysaccharides supplementation reduced diarrhea incidence in calves by modulating gut microbiota, increasing beneficial bacteria, and reducing pathogenic populations. They also regulated immune and inflammatory pathways, alleviating intestinal inflammation, with sea weeds showing greater effectiveness than alfalfa polysaccharides.	[78]
12	Organo sulfur compounds from garlic	Buffalo	2% of dry matter and 112 d	Reduced methane emissions and increased milk yield without altering digestibility or milk composition.	[79]

**Table 3:** The major biological effects of plant bioactive compounds in pigs

S.No	Plant bioactive compounds	Species	Doses	Biological effects	References
1	Quercetin	Growing pigs	0.1% of basal diet and 28 d	Improved growth, nutrient digestibility, and immune responses in LPS-challenged pigs, indicating immunomodulatory and growth-promoting effects.	[25]
2	Puerarin	Piglets	Dosage of 0.5 mg/kg body weight	Enhanced intestinal antioxidant defenses in PEDV-challenged piglets, increasing SOD and GSH-Px and reducing H_2_O_2_ and MDA.	[26]
3	Soy isoflavone	Weaned piglets	40 mg/kg of basal diet and 14 d	Enhanced immune function, reduced diarrhea incidence, and decreased circulating endotoxin levels in LPS-challenged piglets.	[83]
4	Citrus flavonoids	Weaned piglets	80 mg/kg of basal diet and 28 d	Flavonoid supplementation improved piglet growth and intestinal health potentially through modulation of the TLR2/NF-κB signaling pathway. This intervention was associated with a reshaping of the gut microbiota, characterized by increased relative abundances of Bacteroidetes and Prevotella, alongside reductions in Tenericutes and Clostridiales, collectively indicative of a more balanced intestinal microbial community.	[87]
5	Resveratrol	Weaned piglets	300 mg/kg of basal diet and 28 d	Reduced deoxynivalenol-induced intestinal inflammation and oxidative stress, enhancing microbial balance and intestinal barrier integrity in piglets.	[84]
6	Curcumin	Weaned piglets	300 mg/kg and 400 mg/kg or 300 mg/kg or 200 mg/kg of basel diet and 14–28 d	Increased IL-10 expression and secretory IgA, while reducing TLR4, TNF-α, IL-1β expression and intestinal damage markers. Also it alleviated oxidative stress and protected the structural integrity and reduced the intestinal copy number of pathogenic *Escherichia coli*, downregulated the TLR4 signaling pathway.	[88,90]
7	Curcumin	Growing pigs	200 mg/kg of basel diet and 26 to 115 d	Improved leg muscle redox status and meat quality in IUGR offspring by mitigating oxidative stress.	[89]
8	Mixture of chestnut wood tannins and organic acids	Piglets	0.35% additive containing (0.19% and 0.16% of organic acids) 23 to 127 d	The improvement in animal growth performance observed between d 82 and 127 may be associated with a reduction in pathogenic *E. coli* and a concomitant increase in beneficial lactic acid bacteria.	[91]
9	Essential oils containing thymol, cinnamaldehyde, and carvacrol	Weaning pigs and growing pigs	0.01% of basel diet and 35 d	The treatment improved overall animal performance and reduced the incidence of diarrhea, potentially through coordinated effects on immune function, intestinal microbial balance, and nutrient digestibility.	[93]
10	Achyranthes bidentata polysaccharides	Weaned piglets	800 mg/kg and 1,600 mg/kg of basel diet and 28 d	The intervention improved mineral homeostasis, hepatic function, lipid metabolism, immune responses, and resilience to oxidative stress in weaned piglets.	[97]

LPS, lipopolysaccharide; PEDV, porcine epidemic diarrhea virus; SOD, superoxide dismutase; GSH-Px, glutathione peroxidase; MDA, malondialdehyde; TLR2, toll-like receptor 2; NF-κB, nuclear factor kappa B; IL-10, interleukin-10; IgA, immunoglobulin A; TLR4, toll-like receptor 4; TNF-α, tumor necrosis factor-alpha; IL-1β, interleukin-1 beta; IUGR, intrauterine growth restriction.
